# Environmental Health Assessment of Deltamethrin in a Malarious Area of Mexico: Environmental Persistence, Toxicokinetics, and Genotoxicity in Exposed Children

**DOI:** 10.1289/ehp.7652

**Published:** 2005-02-25

**Authors:** María D. Ortiz-Pérez, Arturo Torres-Dosal, Lilia E. Batres, Olga D. López-Guzmán, M. Grimaldo, C. Carranza, Iván N. Pérez-Maldonado, Flavio Martínez, José Pérez-Urizar, Fernando Díaz-Barriga

**Affiliations:** ^1^Facultad de Medicina,; ^2^Unidad Pediátrica Ambiental de la Facultad de Medicina, and; ^3^Facultad de Ciencias Químicas, Universidad Autónoma, San Luis Potosí, México

**Keywords:** children’s health, deltamethrin, genotoxicity, pyrethroids, soil pollution, toxicokinetics

## Abstract

We reported previously that children are exposed to deltamethrin in malarious areas. In the present work we explored the levels of this insecticide in soil samples and also obtained relevant toxico-kinetic data of deltamethrin in exposed children. Results show that, after spraying, indoor levels of deltamethrin in soil samples were higher than outdoor levels. The mean half-life estimated with these data was 15.5 days for outdoor samples and 15.4 days for indoor samples. Children’s exposure to deltamethrin was assessed using as biomarkers the urinary concentrations of the metabolites 3-phenoxybenzoic acid (3-PBA) and *cis*-3-(2,2-dibromovinyl)-2,2-dimethylcyclopropane-1-carboxylic acid (Br_2_CA). The mean level of both biomarkers reached a peak within the first 24 hr postexposure; 6 months after the initial exposure, urinary levels of 3-PBA and Br_2_CA were found at levels observed before exposure. Approximately 91% of the total 3-PBA or Br_2_CA was excreted during the first 3 days after exposure. Therefore, we estimated a half-life for this period, the values for 3-PBA and Br_2_CA being almost identical (13.5 vs. 14.5 hr). Finally, considering reports about the genotoxicity of deltamethrin, we assessed DNA damage in children before and 24 hr after indoor spraying of deltamethrin; we found no differences in the comet assay end points. In conclusion, we observed exposure to deltamethrin in children, but we did not find any relationship between soil concentrations of deltamethrin and urinary levels of the metabolites. At least for genotoxicity, the exposed children appeared not to be at risk.

Indoor spraying of insecticides has been the main strategy for malaria vector control in Mexico. Until the year 2000, DDT (dichloro-diphenyltricholoroethane) was used in this program; since then, deltamethrin has been the insecticide selected for indoor spraying of dwellings in malarious areas (Chanon et al. 2003). Deltamethrin is one of the insecticides recommended by the World Health Organization (WHO) for indoor spraying (WHO 2001) and is one of the insecticides being used for treatment of mosquito nets (Barlow et al. 2001). To maintain coverage in Africa alone, 50 million nets a year are needed (WHO 2003); thus, we can conclude that numerous individuals are being exposed to deltamethrin. However, the number of exposed individuals is larger if we consider that this pyrethroid is also used in agriculture and in household insecticides (EXTOXNET 2003).

Deltamethrin is a type II pyrethroid insecticide that kills insects on contact and through digestion (EXTOXNET 2003). Although deltamethrin appears to be the most persistent pyrethroid in commercial use, laboratory and field studies suggest that this insecticide degrades faster than many of the persistent organochlorides, including DDT. For example, after initial application of 1 mg/kg deltamethrin in a mineral soil, 52% of that concentration was recovered after an 8-week incubation [Chapman and Harris 1981; Chapman et al. 1981; International Programme on Chemical Safety (IPCS) 1990]. However, in an organic soil the percentage of recovery increased to 74% (Chapman and Harris 1981; Chapman et al. 1981; IPCS 1990).

Ingestion of treated soil particles and contact with sprayed surfaces can be important pathways of exposure for children living in dwellings exposed to deltamethrin. However, inhalation of particles can also be relevant if children enter the sprayed room just after deltamethrin application. Absorption of pyrethroids through lungs, gastrointestinal tract, and skin has been observed in humans [Agency for Toxic Substances and Disease Registry (ATSDR) 2001]. It appears that pyrethroids are rapidly absorbed after inhalation, based on the appearance of urinary metabolites within 30 min of exposure (ATSDR 2001). In turn, oral absorption seems to be more important than dermal. For example, up to 63% of the administered dose was recovered in male volunteers after oral exposure to a type II pyrethroid (Eadsforth et al. 1998; Woollen et al. 1992), whereas only 1.8% of the same pyrethroid applied to volunteers was absorbed through skin (Woollen et al. 1992). Absorption, distribution, and excretion have been studied in three male human volunteers given ^14^C-radiolabeled deltamethrin as a single 3.0-mg dose orally (IPCS 1990). Plasma concentrations were maximal 1–2 hr after administration with an apparent elimination half-life in plasma of 10–11.5 hr (IPCS 1990). Over 5 days, 10–26% of the dose was eliminated via feces and 51–59% via urine; 90% of urinary excretion was within the first 24 hr (IPCS 1990). Urinary half-life of 10–13.5 hr was consistent with plasma half-life (IPCS 1990).

Although acute effects of deltamethrin, including nervous system effects and allergic reactions, have been described in exposed populations, limited information regarding chronic effects in humans is available (ATSDR 2001; EXTOXNET 2003; IPCS 1990). Therefore, two interesting *in vitro* effects—deltamethrin-elicited neuronal apoptosis, possibly mediated by nitric oxide synthase (Wu et al. 2003), and deltamethrin-induced DNA damage, as revealed by the comet assay (Villarini et al. 1998)—merit more studies in exposed populations. In such studies, measurement of urinary metabolites may serve as a useful marker of exposure. In fact, for delta-methrin, 3-phenoxybenzoic acid (3-PBA) and *cis*-3-(2,2-dibromovinyl)-2,2-dimethylcyclo-propane-1-carboxylic acid (Br_2_CA) have been used as biomarkers either in urban populations (Heudorf and Angerer 2001) or in exposed workers (Tuomainen et al. 1996).

Many of the effects caused by deltamethrin have been reported in exposed workers; however, considering that the capability of children for detoxification of pyrethroid compounds through metabolic pathways may be different from that of adults, and taking into account that this difference could result in an increased distribution of unmetabolized pyrethroids to the central nervous system (ATSDR 2001), it is important to study the exposure of children to this insecticide. We have previously reported that, in malarious areas, children are exposed to deltamethrin (Yáñez et al. 2002). In that study, we suggested, as a working hypothesis, that children are exposed either by ingesting contaminated soil or by having contact with sprayed areas (in the tropics children normally play close to the walls because these are the areas most protected from the sun). In the present work, we explored this hypothesis further, and we also include relevant data with regard to the toxicokinetics of deltamethrin in exposed children.

## Materials and Methods

### Population.

Participants were unpaid volunteers selected from four malarious communities located in the state of San Luis Potosí, Mexico. Children were selected from those living in 16 sprayed dwellings. Twenty-four girls and eight boys, 3–12 years of age, were studied. All the children lived at their same address for the duration of this study. After informed consent was obtained, a questionnaire was administered and urine samples were taken. The questionnaire registered socio-demographic characteristics, occupation of parents, and food habits. Deltamethrin was sprayed on the walls and ceilings of residences as a wettable powder (2.5%) at a dose of 25 mg/m^2^. Urine samples were collected in sealable plastic bottles and stored in the deep freezer until analysis. The samples were obtained at day 0 (previous to exposure to deltamethrin spraying) and at days 1, 2, 3, 7, 15, 30, 45, and 180 after exposure. During the first 2 days, seven samples were collected approximately 6 hr apart. On other days, only first void samples were obtained.

### Environmental samples.

All of the 16 dwellings had dirt floors. Surface soil samples indoors and outdoors (1–3 cm) were collected in aluminum foil. Two composite samples (one indoor and one outdoor) were obtained from each dwelling. Composite samples were generated with four individual samples collected in each corner of the main room (the one in which the children sleep and play). Samples were obtained at day 0 (previous to the spraying) and at days 1, 8, 15, 30, 45, 60, and 180 after spraying the room with deltamethrin. Samples were transported to the laboratory, dried at 50°C, sieved, and kept under refrigeration (4°C) until analysis.

### Urinary 3-PBA and Br_2_CA analysis.

We quantified 3-PBA and and Br_2_CA following the method described by Angerer and Ritter (1997). Under our conditions, the method detection limits were 0.58 μg/L for 3-PBA and 0.185 μg/L for Br_2_CA. The repeatability precision was 5.5% and 6.13% for 3-PBA and Br_2_CA, respectively. Intralaboratory reproducibility was 1.43% for 3-PBA and 2.58% for Br_2_CA. Recoveries averaged 117%.

### Deltamethrin analysis in soil.

Soil samples (1 g) were microwave extracted in 15 mL acetone:hexane (1:1) using Mars 5-MES 1000 (CEM Corporation, Matthews, NC, USA) equipment. The microwave conditions were as follows: power, 100%; extraction temperature, 100°C; extraction time, 30 min. After the extraction, samples were filtered and evaporated close to 0.1 mL, using a gentle stream of nitrogen. Analyses were performed by gas chromatography using a Hewlett-Packard model 6890 chromatograph (Agilent Technologies, Palo Alto, CA, USA) with an autosampler and a split/splitless injector operating in the splitless mode. The inlet purge off time was 2 min. The operating temperature for the injector was 280°C. Separation was carried out on an HP-5 column (5% phenyl-methylpolysiloxane; Hewlett Packard, Agilent Technologies), 60 m × 0.25 mm inner diameter, 0.25 μm film thickness. Column temperatures were as follows: initially 150°C held for 2 min, raised at a rate of 15°C/min to 300°C, and held at this temperature for 20 min. The transfer line temperature was maintained at 290°C. Helium was used as the carrier gas at a linear velocity of 1.1 mL/min. Injection volume was 2.0 μL. The quantitative analysis of deltamethrin was performed by selected ion monitoring, using mass spectrometry (HP 5973 mass spectrometer; Hewlett-Packard, Agilent Technologies). The characteristic ions were 181 and 253. Under these conditions and using data generated by seven replicates near the lowest concentration attainable at the calibration curve, the method detection limit for deltamethrin was 0.2 mg/kg and the quantification limit was 0.63 mg/kg. The between-assay variation co-efficient was 7 ± 2%. Recovery averaged 85 ± 4.4%. The linear range used for this determination was 0.6 at 15 mg/kg. For the half-life estimation of deltamethrin in soil, we followed a first-order degradation model as described by Hill (1983).

### DNA damage.

We evaluated this parameter using the comet assay following the method reported by Singh et al. (1988). Details of the method have been described previously (Yáñez et al. 2004). The blood samples were obtained in 28 children at day 0 (before the deltamethrin was sprayed) and 24 hr after spraying.

### Toxicokinetic parameters.

Individual time courses of urinary 3-PBA and Br_2_CA rate of excretion, expressed as creatinine-corrected values, as well as cumulative curves of metabolite excretion, were constructed. We estimated peak rate excretion, percentage of urinary excreted metabolites, and apparent half-life (by regression analysis of log-transformed) by non-compartmental methods, using the software WinNonLin Pro R.2.1 (Pharsight, Mountain View, CA, USA).

### Statistical analysis.

Data were transformed logarithmically to adjust to a normal distribution. A paired *t*-test, pairing the indoor and outdoor levels at each location, was used to assess the significance of indoor versus outdoor soil deltamethrin concentrations. Differences in half-life of deltamethrin in surface soil samples were analyzed using analysis of variance followed by a comparison analysis using the Tukey procedure. A *p* < 0.05 value was considered to be statistically significant. A *t*-test was used to studied differences between 3-PBA and Br_2_CA in urinary concentrations, half-life, and cumulative excreted concentration (CEC). Analysis of paired Student *t*-test was used to test differences in the comet assay. The correlation analyses were done with log-transformed data. For all statistical analyses, we used JMP IN software (version 5.0.1.2; SAS Institute, Inc., Cary, NC, USA).

## Results

**Figure 1:**
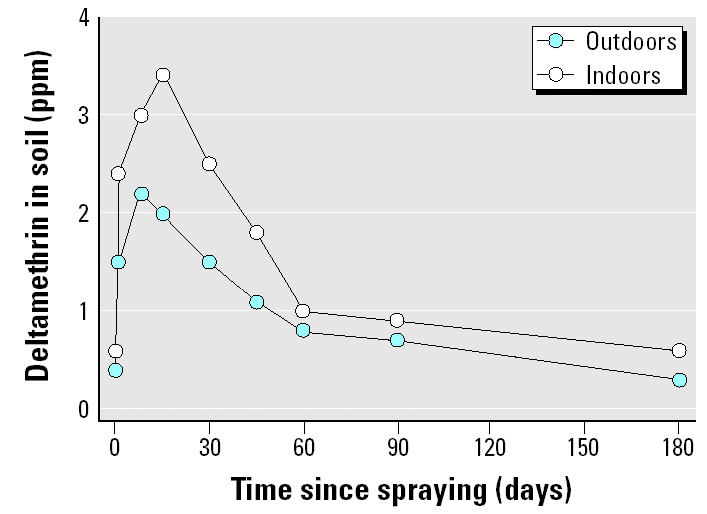
Outdoor and indoor time course of delta-methrin surface soil levels: mean concentration of all the sampled soils. Deltamethrin was sprayed on day 1.

We quantified the levels of deltamethrin in surface soil samples in four communities: Tancuime, El Chuche, El Naranjal, and El Topo. These samples were obtained before and after spraying the insecticide. Results showed that indoor levels were higher than outdoor levels (*p* < 0.001) (Table 1). The maximum concentration in both environments (outdoors and indoors) was registered between 8 and 15 days after spraying (Table 1, Figure 1). Background levels were recovered on different days in the four communities studied in this work, but at 60–90 days after the application, deltamethrin levels were close to the background levels in all the communities (Table 1). The mean half-lives estimated with these data were 15.5 days for outdoor samples and 15.4 days for indoor samples; however, different half-lives were estimated in the four communities (Table 2). Moreover, although in general the differences in deltamethrin half-lives in soil between indoor and outdoor environments were not statistically significant, it is worth noting that the organic carbon content outdoors (3.1% ± 1.2) was significantly higher than that indoors (2.2% ± 0.8, *p* < 0.05). No differences among communities were found in soil carbon content.

## Discussion

Deltamethrin is being used for the control of malaria in different countries, and indoor spraying may make soil contamination a pathway of exposure for children. In this work, the highest concentration found in soil was 8.9 mg/kg (corresponding to an indoor sample collected in the community of El Naranjal; data not shown), a concentration 32 times lower than the environmental guideline for soil calculated using 0.01 mg/kg/day as an acceptable daily intake for deltamethrin (WHO 2002), 10 kg of body weight, and 350 mg/day of soil ingestion (Díaz-Barriga et al. 1997). Thus, for the concentrations of deltamethrin sprayed in the communities studied in this work, soil ingestion might not be an important pathway of exposure for children.

Indoor deltamethrin soil levels were higher than outdoor concentrations; however, half-lives were very similar. Taking into account that photolysis and biodegradation have been reported in soils treated with deltamethrin, a higher degradation rate for outdoor soils would be expected. However, a possible explanation for this result may be that the content of organic carbon outdoors was higher than indoors. It has been reported that 8 days after treatment, more deltamethrin was recovered from an organic soil than from a sandy soil (Chapman and Harris 1981). The interaction of deltamethrin with soil would decrease the bioavailability and thus the biodegradation of this insecticide.

Although a half-life of 6.8 weeks has been reported for deltamethrin in soil under field conditions, a half-life of 4.8 weeks was reported for indoor experiments (Hill 1983). In this study, we report half-lives of 2.22 weeks (outdoors) and 2.20 weeks (indoors). However, the two studies cannot be compared, because in our study meteorologic conditions (e.g., ambient temperature, humidity, rain) were not controlled during the 6 months of the study. Also, in the present work, different half-lives were found among communities; however, considering the low number of soil samples studied in some of them, it is not possible to postulate further conclusions.

Taking into account that deltamethrin was sprayed in homes, it was important to follow the exposure in children by analyzing urinary metabolites of this insecticide. Different studies have shown that 3-PBA and Br_2_CA can be used as biomarkers of exposure for deltamethrin (Heudorf and Angerer 2001; Tuomainen et al. 1996). In our study, urinary concentrations of both metabolites increased after exposure (Table 3), and a significant correlation was found between them. However, the concentrations of Br_2_CA were higher than those of 3-PBA. Although data for humans are limited, in animals the metabolism of deltamethrin includes oxidative attacks at several sites, and conjugation reactions to produce a complex array of primary and secondary water-soluble metabolites (ATSDR 2001; IPCS 1990). Our results indicate that the metabolic pathway of 3-PBA may include more degradation steps than does that of Br_2_CA. Therefore, and taking into account that 3-PBA is not specific for deltamethrin exposure (Heudorf and Angerer 2001), we recommend using Br_2_CA as a biomarker of exposure to deltamethrin.

Few studies have reported exposure to deltamethrin in children (Heudorf and Angerer 2001; Heudorf et al. 2004; Yáñez et al. 2002). Our results in this study (urinary concentration of 3-PBA) are similar to those reported by our group in the state of Oaxaca 2–3 days after exposure (Yáñez et al. 2002). Together, both studies show that children living in malarious areas where deltamethrin is sprayed for vector control are more exposed than are children in the general population (Heudorf and Angerer 2001; Heudorf et al. 2004; Yáñez et al. 2002).

Taking into consideration that 91% of the total 3-PBA or Br_2_CA was excreted during the first 3 days after exposure, an apparent half-life for this period was estimated. The results for 3-PBA (13.5 hr) and Br_2_CA (14.5 hr) were in the range of what was reported in three young male human volunteers who received a single dose of 3 mg of ^14^C-deltamethrin. In that study, the apparent half-life of urinary excretion was 10.0–13.5 hr, and 90% of this radioactivity was excreted during the 24 hr after absorption (IPCS 1990).

In our study, considering the concentration of metabolites in urine, the main exposure takes place during the first 3 days after spraying; however, detection of metabolites was possible until 45 days after spraying. The presence of metabolites in all this period may reflect a constant exposure (i.e., due to the presence of deltamethrin in soil). Thus, a second apparent half-life was estimated for the 7- to 45-day postexposure period. In contrast to the initial half-life (estimated for the first 3 days postexposure), in this second half-life we observed a significant difference between the results obtained with 3-PBA and Br_2_CA; the second half-life of 3-PBA (288 hr) was higher than that estimated with Br_2_CA levels (197 hr) (Table 4). This result would reflect that the metabolic pathways for the two metabolites are different.

We did not find any correlation between the half-lives with sex or age. However, we did observe a significant inverse correlation between the first half-life and the CEC. Considering that CEC is an indicator of the magnitude of exposure, this result is important because it implies that the metabolism of deltamethrin in humans may be autoinducible. In rats, evidence of development of tolerance on repeated dosing with deltamethrin suggests that the compound induces its own metabolism (Barlow et al. 2001).

We also found an inverse correlation of CEC with age. This result indicates that exposure is related to time at home. Thus, for a risk reduction program, it would be important to identify those pathways of exposure in the home environment. In this regard, three routes of exposure are important for deltamethrin: inhalation, ingestion (in this case of soil particles), and dermal absorption. Considering that the maximum concentration of deltamethrin in soil was not observed until 8–15 hr post-application (Table 1), that deltamethrin content in soils remains almost constant during the first month after spraying (Table 1), and that urinary metabolites content increases from the first day but that it abruptly decreases by day 15 postspraying, the soil pathway could be dismissed as the most important pathway. In contrast, it has been reported that the concentrations of cypermethrin (another halogenated type II pyrethroid), detected in indoor air of vacant dormitory rooms after its application for cockroach control, were 18.2, 8.5, and 3.0 μg/m^3^ at 0, 7, and 28 days postapplication, respectively (Wright et al. 1993). Assuming that these data can be taken as a good example for deltamethrin, we can argue that inhalation may be an important pathway of exposure during the first days postspraying. In regard to dermal absorption, we have to take into account that, in general, the absorption of pyrethroids through skin is limited; for example, it was estimated that 1.8% of the applied dose was absorbed in volunteers after dermal application of cypermethrin (Woollen et al. 1992). In conclusion, inhalation during the first hours or days postapplication may be considered the main pathway of exposure, leaving soil ingestion and dermal exposure as secondary routes.

Diet can be excluded as a source of delta-methrin in the studied population because the urinary levels of the metabolites found in all children before spraying were lower than the detection limit of the analytical method.

Children in this study were exposed to deltamethrin; thus, we decided to assess DNA damage in them, before and after exposure. Results were negative because no differences were observed. In the literature, DNA damage elicited by deltamethrin has been reported in human peripheral blood leukocytes treated *in vitro*; however, the minimum significant dose of the insecticide in that study was 100 μg/mL (Villarini et al. 1998). Therefore, we can speculate that deltamethrin blood concentration of the studied children was < 100 μg/mL.

Deltamethrin has been related to a variety of dermal and neurologic symptoms, and although in this work we did not investigate these symptoms in detail, in talking to the parents we learned that no effects in children were related to this insecticide. However, in other malarious communities of Mexico (Pérez-Maldonado I, Díaz-Barriga F, unpublished observations), the children complain about redness, burning sensation, and itching. In this regard, it is important to take into account that, on the basis of human biomonitoring data, it has been difficult to relate exposure with effects. For example, dermal or neurologic symptoms in sprayers exposed to deltamethrin have showed no significant correlation with urinary metabolites excretion (He et al. 1991; Zhang et al. 1991).

In this study, we demonstrated that the health risk for children exposed to delta-methrin in malarious areas can be reduced if precautions are taken, at least during the first 24 hr after spraying. For example, the access of children to sprayed areas has to be limited; furthermore, no foodstuffs should remain in the area during spraying or until 24 hr after. Cooking can be done only after the first day and just after cleaning all the cooking areas (e.g., tables, chairs, rustic oven). It is important to remember that ambient conditions may modify the exposure (i.e., increased volatilization of deltamethrin); furthermore, keeping children’s behavior under observation is a good practice to control soil ingestion and dermal contact with sprayed areas. It is important to consider that although the levels and the effects described in this article may suggest a minimum risk for children, in other communities the situation could be different; therefore, it is important to institute surveillance programs in those communities treated with pesticides. Moreover, studies are needed to assess the exposure to deltamethrin when other formulations are used. For example, a 25% water-dispersible granule formulation of deltamethrin has been recommended by WHO for use for indoor residual spray in malaria vector control programs (WHO 2002). Finally, exposure assessment programs are imperative for communities where a pyrethroid is used for the control of malaria and an organophosphate is used for the control of dengue. An interaction between these types of insecticides has been reported in the literature (Ortíz et al. 1995).

## Figures and Tables

**Figure 2:**
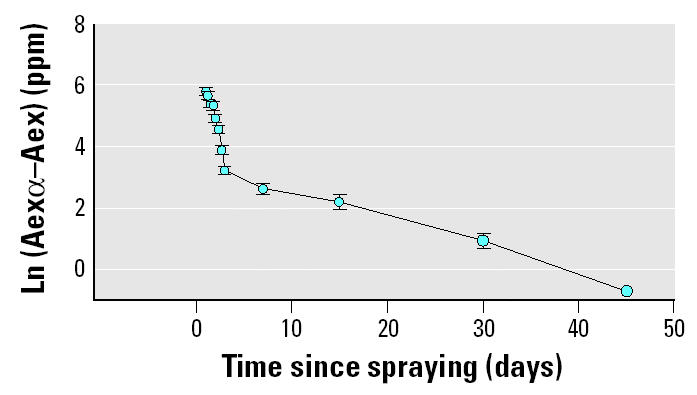
Time course of the logarithm of the amount to be excreted showing the biexponential elimination kinetics of deltamethrin metabolite Br_2_CA in all children. Abbreviations: Aex, delta-methrin’s metabolite excreted at a particular time; Aexα, total of deltamethrin’s metabolite excreted during entire period of sampling. The mean ± SE are shown for each day.

**Table 1:** Deltamethrin mean levels (mg/kg) in surface soil samples before and after spraying.

	Days	No.	Outdoor	Indoor
Tancuime	0	9	0.4	0.6
	1	9	1.4	2.2
	8	9	3.0	3.7
	15	9	1.7	2.6
	30	9	1.0	2.0
	45	9	0.9	1.6
	60	9	0.6	0.8
	180	9	0.3	0.4
El Naranjal	0	3	0.4	1.4
	1	3	1.7	4.4
	8	3	0.8	3.3
	15	3	3.2	7.0
	30	3	2.4	4.4
	45	2	1.6	4.4
	60	2	1.3	1.8
	90	2	0.6	0.6
	180	2	0.4	0.6
El Chuche	0	3	0.3	0.4
	1	3	0.8	1.2
	8	3	0.6	0.8
	15	3	1.2	2.4
	30	3	1.2	2.0
	45	3	1.1	1.1
	60	3	0.4	0.9
	90	3	0.5	0.5
	180	3	0.5	0.6
El Topo	0	1	0.3	0.3
	1	1	3.4	1.1
	8	1	4.4	4.3
	15	1	3.3	3.2
	30	1	3.9	4.4
	45	1	1.9	1.2
	60	1	3.3	1.6
	90	1	1.7	0.3
	180	1	0.3	0.3

Indoor versus outdoor *p* < 0.001.

**Table 2:** Half-life of deltamethrin in surface soil samples (days).

Community	No.	Mean ± SD	Range
Tancuime			
Outdoor	9	13.4 ± 1.7	10.9–16.4
Indoor	9	13.2 ± 2.6	9.4–17.5
El Chuche			
Outdoor	3	25.2 ± 6.0	20.2–31.9
Indoor	3	21.5 ± 6.3	16.2–28.5
El Naranjal			
Outdoor	2	18.4 ± 2.1	16.8–19.9
Indoor	2	17.1 ± 2.2	15.5–18.6
El Topo			
Outdoor	1	15.1	—
Indoor	1	14.3	—
Total			
Outdoor	15	15.5 ± 5.5	10.9–31.9
Indoor	15	15.4 ± 4.6	9.4–28.5

Indoor, no differences among communities. Half-lives of outdoor samples between El Naranjal and Tancuime versus El Chuche are significantly different, *p* < 0.05.

**Table 3:** Urinary deltamethrin metabolites in children living in sprayed residences.

			3-PBA		Br_2_CA	
	Days	No.	Mean	Range	Mean	Range
Tancuime	0.00	22	ND	—	ND	—
	0.25	22	15.5	3–50	24.0	ND–93
	0.50	22	26.9	5–71	50.0	6–134
	0.75	22	35.2	9–135	83.5	20–226
	1.00	22	34.8	5–148	44.1	3–200
	1.33	22	22.5	6–95	63.0	8–350
	1.66	22	17.5	ND–109	65.0	ND–275
	2.00	22	27.3	4–106	60.0	15–368
	3.00	22	8.1	ND–20	28.7	ND–150
	7.00	22	4.6	ND–30	12.4	3–45
	15.00	22	1.5	ND–6	6.0	ND–23
	30.00	22	1.9	ND–4	9.3	ND–27
	45.00	22	3.5	ND–10	6.3	ND–43
	180.00	22	ND	—	ND	—
El Naranjal	0.00	3	ND	—	ND	—
	0.25	3	25.1	19–37	90.4	70–122
	0.50	3	56.3	40–71	120	28–271
	0.75	2	37.8	29–46	136	55–217
	1.00	1	25.8	—	11.1	—
	1.33	3	31.5	17–41	55.6	22–80
	1.66	2	17.5	13–22	93.8	35–182
	2.00	3	27.7	20–35	42.1	24–71
	3.00	2	5.7	2–12	2.4	ND–6
	7.00	2	5.8	5–7	25.6	24–26
	15.00	2	1.0	ND–3	3.6	ND–4
	30.00	2	3.6	3.6	16.9	15–18
	45.00	2	7.4	3–11	ND	—
	180.00	2	ND	—	ND	—
El Chuche	0.00	5	ND	—	ND	—
	0.25	5	11.8	4–26	17.6	3–50
	0.50	5	13.0	5–20	27.0	10–56
	0.75	5	21.3	13–31	57.2	30–95
	1.00	5	21.8	13–37	19.0	9–34
	1.33	5	20.1	9–27	50.0	14–140
	1.66	5	7.4	ND–20	46.0	ND–180
	2.00	5	16.9	11–22	31.4	19–59
	3.00	5	7.4	ND–26	20.1	ND–43
	7.00	5	5.2	3–8.2	13.4	6–21
	15.00	5	1.8	ND–5	4.4	ND–11
	30.00	5	1.8	ND–4.3	8.6	1–10
	45.00	5	3.0	ND–7	7.1	ND–18
	180.00	5	ND	—	ND	—
El Topo	0.00	2	ND	—	ND	—
	0.25	2	5.8	4–7	7.0	5–9
	0.50	2	7.6	5–10	11.0	9–13
	0.75	2	9.9	8–11	31.2	20–43
	1.00	2	10.6	8–13	32.0	16–49
	1.33	2	9.8	8–12	24.0	13–35
	1.66	2	4.2	3–5	18.6	4–34
	2.00	2	12.7	8–17	40.5	36–45
	3.00	2	6.1	5–7	10.8	4–17
	7.00	2	5.5	4–7	10.3	10–11
	15.00	2	1.5	1–2	7.3	4–10.6
	30.00	2	2.5	2–3	5.0	1–9
	45.00	1	4	—	14.3	—
	180.00	2	ND	—	ND	—

ND, not detectable. The analyses were done before and after spraying (days). Results are μg/g creatinine. Using a paired *t*-test, differences between both metabolites were found to be significant (*p* < 0.05) when using the information of all the children in the study.

**Table 4:** Toxicokinetic parameters in children exposed to deltamethrin.

	3-PBA	Br_2_CA
*T*_1/2_ (first 3 days)	13.5 ± 3.7	14.5 ± 4.0
*T*_1/2_ (7–45 days)	288.8 ± 98.9*	197.5 ± 78.8
CEC (μg/g creatinine)	172.1 ± 98.3*	409.7 ± 254.2

*T*_1/2_, half-life (hours). Values are arithmetic mean ± SD.

* p < 0.05.

**Table 5:** DNA damage in children exposed to delta-methrin.

End point	Sample	Mean ± SD
Tail moment	Before spraying	9.1 ± 3.2
	24 hr after spraying	9.1 ± 3.0
Tail length	Before spraying	43.2 ± 12.7
	24 hr after spraying	39.2 ± 9.6

DNA damage was assessed by the comet assay, *n* = 28.
